# Generative artificial intelligence in physiotherapy education: great potential amidst challenges- a qualitative interview study

**DOI:** 10.1186/s12909-025-07106-w

**Published:** 2025-04-24

**Authors:** Yvonne Lindbäck, Karin Schröder, Torkel Engström, Karin Valeskog, Sofi Sonesson

**Affiliations:** Unit of Physiotherapy, Department of Health, Medicine and Caring Sciences, Linköping University, Linköping, Sweden

**Keywords:** Generative artificial intelligence, Health and medical education, Physiotherapy, Focus group interviews, Qualitative method, Student perspective

## Abstract

**Background:**

Generative Artificial Intelligence (GAI) has significantly impacted education at all levels, including health professional education. Understanding students’ experiences is essential to enhancing AI literacy, adapting education to GAI, and implementing GAI technology. Therefore, the aim was to explore physiotherapy students’ experiences of and thoughts on GAI in their education, and its potential implications for their future careers in healthcare.

**Methods:**

Qualitative descriptive design. Focus groups were conducted, using a semi-structured interview guide, at the Physiotherapy program at Linköping University, Sweden, from March to April 2024. The 15 students were organized into three focus groups, one for each education year. The data was analyzed using inductive content analysis.

**Results:**

An overarching theme “GAI—Great potential if navigating the challenges” emerged from three categories: 1) “Areas of GAI use in the learning process”: Students viewed GAI as a tool for introduction and inspiration, assimilating course content and enhancing clinical reasoning and problem-solving; 2) “Optimizing GAI use in education”: Students found GAI to be timesaving, tailored, and as a virtual study partner and teacher. They discussed the pros and cons of learning, concerns on permitted GAI usage, the need for a critical approach, and how individual experiences and interests influenced their interactions with GAI; 3) “Future with GAI in education and profession”: Students believed future GAI would be more reliable, use subject-specific GAI models and enhance health care delivery, but also pose risks related to profit motives and knowledge gaps.

**Conclusion:**

Physiotherapy students found GAI beneficial for learning and clinical reasoning but expressed concerns about its impact on learning quality. They emphasized the importance of a critical approach when using GAI and the need for organizational support, including supporting permitted GAI use. Students believed that future advanced GAI models could provide accurate and reliable educational tools and healthcare tools supporting documentation and evidence-based decision-making. However, potential risks include business profit motives and knowledge gaps. Navigating these challenges is essential to fully leveraging GAI’s benefits in education and physiotherapy practice. Therefore, fostering a critical approach and ensuring robust organizational support is crucial for maximizing the positive impact of GAI in physiotherapy.

**Supplementary Information:**

The online version contains supplementary material available at 10.1186/s12909-025-07106-w.

## Introduction

The exponential growth of Generative Artificial Intelligence (GAI) and Large Language Models (LLMs) has significantly impacted teaching and learning at all educational levels [1]. In higher education, GAI can provide accurate responses, enhance texts, summarize research, create virtual patient scenarios, and explain concepts and key findings [2–4]. From a student perspective, GAI serves as a versatile teaching aid, simplifying and summarizing text to facilitate understanding [5]. Improved AI literacy and sustainable integration can foster creativity and self-efficacy, thereby enhancing learning performance and academic achievements [6]. However, from a teacher assessment perspective, distinguishing AI-generated texts from independently written ones is challenging [7], and no current tool can reliably make this distinction [1]. This raises concerns about assessment reliability and validity, academic integrity, and whether assessments accurately reflect students’ knowledge and skills [1, 7]. Beyond the risk of cheating, GAI may hinder the development of critical thinking, problem-solving skills and comprehensive educational development [8]. Additionally, GAI poses risks of incorrect or misleading information because of how it processes existing data [9]. Since GAI generates text based on probabilistic word sequences, it can mix true and fabricated content, potentially leading to students’ knowledge being based on incorrect facts [2, 10].

The risks and benefits of students using GAI in their education can be related to student agency, which involves actively mastering learning by making responsible choices, setting goals, and reflecting on the learning process. Student agency is key for success in higher education and includes self-regulated learning, self-efficacy, intentionality, forethought, and self-awareness [11]. Self-regulation is a process in which the students transform their mental abilities to achieve academic goals through personal strategies [12]. Students with high agency show greater motivation to learn, define learning objectives, and develop lifelong learning skills [11]. They use a deep learning approach with GAI, actively engaging in critically assessing AI-generated outputs, evaluating their validity, and reflecting on their contribution to a broader understanding of the subject. This involves integrating AI-generated content with prior knowledge and personal experiences to construct a meaningful comprehension. In contrast, students with low agency tend to accept GAI outputs uncritically, without evaluating their reliability or relevance, leading to superficial knowledge and limited understanding [13]. A survey of Swedish university students revealed that most were supportive of GAI in education but concerned about its impact on education quality and cheating. Additionally, there was uncertainty regarding institutional GAI guidelines, indicating room for improvement [14].

Knowledge in integrating GAI into health professional education is limited. A review in nursing education found that the literature mainly consists of editorials and review articles from the USA, China, Canada, India and the Philippines and primarily involves academic writing, health care simulation, data modelling and personal development [15]. In medical education, studies mostly focus on clinical specialty training and continuing education, while GAI integration in education is expanding, needing consensus on ethical issues and regulation standards [16]. However, there is a significant gap in AI applications in physiotherapy education compared to medical and dental education [17].

Health professional students may use GAI in their future healthcare roles as consumers, translators, or developers, requiring knowledge and skills in technical concepts, validation, critical appraisal, and ethics [18]. Potential uses of AI in physiotherapy practice include developing and training LLMs for administrative tasks, simulated patient interactions, clinical decision-making support, guideline recommendations, and tailored treatments [19]. Furthermore, computer vision and machine learning can accurately monitor home exercise movements [20].

Qualitative studies provide in-depth insights into complex phenomena, encouraging open discussion and exploration [21], which are crucial for implementing GAI in health professional education. Two previous descriptive interview studies showed mixed attitudes toward GAI; medical students and educators found it enhances clinical reasoning [22], while nursing students believed it improves understanding and efficiency but worried it may impede critical thinking and emotional skills [23]. Additionally, in a focus group study, health science students found ChatGPT- 3.5 easy to learn but noted its shortcomings in reliability, accuracy and academic integrity [24]. Qualitative studies on physiotherapy students’ experiences of GAI are lacking. Therefore, this study explores physiotherapy students’ experiences and thoughts on GAI in their education and its potential implications for their future careers in healthcare.

## Method

### Study design

We employed a qualitative descriptive design. The data was collected through focus group interviews and analysed using inductive content analysis, to gain insights into the informants’ experiences of the phenomenon [25]. The COnsolidated criteria for REporting Qualitative research (COREQ) checklist [26] was applied.

### Data collection

The inclusion criteria were students enrolled in the Physiotherapy program at Linköping University, which has 224 students. A purposeful sampling was used to achieve variation in study habits and knowledge levels concerning the phenomenon of interest [25], and the study population was selected to include students in their first, second, and third years of education.

Study participants were recruited through written information and inquiries via the digital learning platform. This was followed by oral and written information about the study and its voluntary nature, presented during course dialogues and teaching sessions. Focus groups were used to facilitate dynamic interactions, leading to a deeper understanding of the topic [27]. Fifteen students were enrolled; all received written information and signed consent forms before the interview. Demographic data including gender, age and other characteristics were also collected (Table 1). The three focus groups included four, five and six students respectively, divided into education year-specific groups to promote conducive conditions for open discussions and high-quality data as the participants were already well acquainted [25]. No additional information was expected after the three focus groups. A semi-structured interview guide (Appendix 1) was designed to be unbiased and non-threatening, with open-ended questions, to explore the students’ experiences [28]. The interview guide was tested through pilot interviews with two individuals outside the project but with relevant experience [29] in university studies and GAI. Minor adjustments in wording were made after the pilot interviews.

**Table 1:** Demographic characteristics of the 15 informants

**Variable**		
Age, median (IQR)	25	(23–30)
Women, n (%)	7	(47)
Work experience, n	15	
Median years (IQR)	3	(1–3)
Previous post-secondary education		
None	8	
0–1 year	4	
1–2 years	1	
More than 2 years	2	
Use of GAI in studying		
Never	1	
Once a month	2	
Once a week	8	
Every day	4	

*IQR* Interquartile range, *GAI* Generative Artificial Intelligence

The interview started by clarifying that we were interested in students’ experiences and thoughts about GAI in their studies. It was emphasized that the focus group content should not be shared and would not affect their teaching or assessments. Participants were encouraged to answer all questions but were not obligated to do so, and they were stimulated to speak freely and from their own experience [30]. Probing questions were used as a complement to gain a deeper understanding [25]. Two authors conducted the focus group interviews; TE was the moderator and YL was the assistant moderator. All authors are teachers in the physiotherapy unit and are known to the students. YL, KS and SS are trained and experienced in performing qualitative interviews, while KV specializes in medical education. The three focus groups were conducted face-to-face on university premises, in March and April 2024. The interviews were audiotaped, lasted 52–60 min, and were transcribed verbatim by TE. Member checking was not conducted.

### Data analysis

The data was analyzed using quality content analysis according to Hsieh and Shannon [31], to identify patterns and themes systematically. Due to limited prior knowledge of the phenomenon studied, an inductive approach was employed [25]. All transcripts were read multiple times, and text sequences consistent with the study aim, i.e. meaning units, were identified and extracted. The meaning units were given codes [31], and subsequently organized into subcategories and categories. Initially, TE and YL independently coded one of the transcribed focus group interviews and discussed meaning units and codes to ensure analytical triangulation [32]. Thereafter, TE coded all transcripts for the two other interviews and proposed a categorization which was then discussed and negotiated between the two authors (TE, YL). There were discussions and suggestions from the other authors in the research team until a consensus was reached, to enhance credibility [25]. The informants and context are described to further improve credibility and validity, and quotations are used to illustrate the result [33]. Each quotation is identified by a number code for the informant. Same periods (…) indicate a pause or that the sentence’s beginning or end is irrelevant and has been omitted. A slash (/) indicates a cut in the text because the information is irrelevant in the context. Parentheses () indicate that words have been omitted. The NVivo version 14 software was used for the analysis.

## Results

Three focus groups were conducted with 15 students, organized into one focus group for each of the three years in the physiotherapy program at Linköping University, Sweden. The informants’ median age was 25 years and there was a variety of use of GAI in their studies. Seven informants were women (47%) (Table 1).

**Fig. 1:**
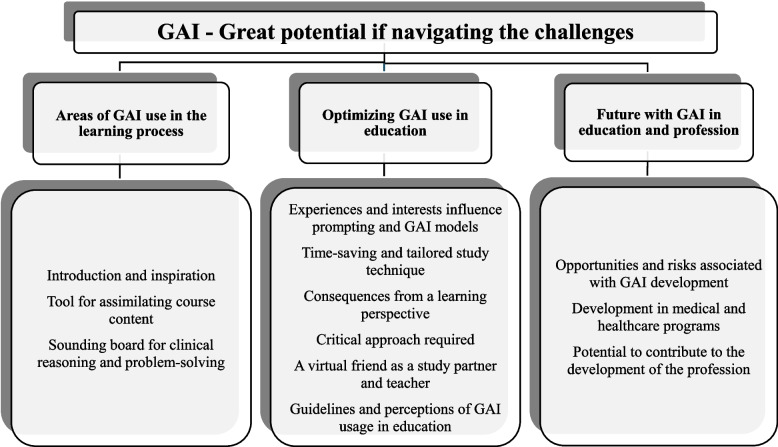
Theme, categories, and subcategories. GAI, Generative artificial intelligence

The analysis revealed an overarching theme and four categories with 16 subcategories. The theme “GAI—Great potential if navigating the challenges”, described students’ different areas of GAI use, advantages and concerns regarding the impact on learning, the need for a critical attitude, and thoughts on the future with GAI. The categories were: 1) “Areas of GAI use in the learning process” 2) “Optimizing GAI use in education” and 3) “Future with GAI in education and profession” (Fig. 1).

### Areas of GAI use in the learning process

#### Introduction and inspiration

The informants described using GAI at the start of exploring a new knowledge area and that GAI provided substantial assistance by offering relevant information, like an encyclopedia. This initial introduction helped develop an overview and sparked further interest in delving deeper into the subject. Further, GAI provided new perspectives that made it easier to identify relevant focus areas and guidance on how to continue to deepen their understanding.

*“…maybe more to explore and try to find out. What should I delve into more, that it can be a support in that way, like for an overview of an area or something like that.”* (3.3).

#### Tool for assimilating course content

GAI was used to simplify and clarify complex concepts, contributing to increased understanding and confirming comprehension of course content. It was employed for translating, processing, and summarizing difficult texts, thus facilitating a more effective assimilation of specialist literature and theoretical content. Students emphasized that while GAI can serve as a valuable tool for enhancing understanding, critical thinking must complement its use. The information generated by GAI was seen primarily as a guide towards deeper understanding.

*“…when there were difficult things I didn’t understand from books or lectures, I could search *via* GAI and then fact-check it with the book.”* (1.1).

#### Sounding board for clinical reasoning and problem-solving

To improve clinical reasoning, GAI was used to generate patient cases for training theoretical knowledge in different diagnoses and disorders, both individually and in groups. It was described as helping to identify and reflect strengths and weaknesses in clinical reasoning when practicing identifying relevant history and examination findings and formulating diagnostic assessments. GAI also supported problem solving by posing questions linked to difficult concepts. It was described as facilitating analysis of problems from different perspectives, suggesting the wording of questions and offering alternative solutions, thereby aiding the learning process.

*“It can be very difficult to understand tests and certain examinations when you don’t have a context to put it into. So, for example, if you ask GAI to give me a patient case of someone having a meniscus injury or similar, you can get a clear case for yourself to which you can apply your knowledge.”* (1.1).

### Optimizing GAI use in education

#### Experiences and interests influence prompting and GAI models

Informants perceived GAI as easy to use in its basic form, allowing them to obtain answers through simple interaction and follow-up questions for more developed responses. The usability of GAI could be further enhanced by following instructional guides for adequate prompting or collaborating with experienced users to understand how variations in interaction could affect responses.

Informants with a prior interest in technology and computer skills were more likely to use GAI regularly. Further, increased self-training was described to enhance their ability to prompt questions and instructions, leading to clearer and more contextually relevant responses.

*“… sometimes you have to be very specific in how you phrase what you’re asking for. Sometimes you get an answer and think, well, that’s not quite what I was looking for. So, you have to ask the question again but phrased differently, and then you might get an answer that’s more what you were looking for.”* (1.2).

Informants selected GAI models based on their prior experiences, economic factors, and computer skills. Limited awareness of various GAI providers, combined with the accessibility and free usage of certain models, often resulted in choosing the most effectively marketed options. As technical functionality improved and experience with GAI grew, informants described being more likely to explore services from different providers. An unawareness of available AI services from the university emerged.

*“I’ve looked at a few different ones, but it’s always that you have to register, or they have a very different interface and are harder to understand, so you don’t bother spending the time on it when you can just use the one you’ve used before.”* (2.4).

#### Time-saving and tailored study techniques

Informants described that GAI tools streamlined various study tasks, freeing time and enabling faster learning progression while reducing the risk of becoming ensnared in less relevant tasks. Faster information retrieval, text processing, and summarizing support led to more efficient study time by prioritizing and deepening knowledge in important areas. Further, GAI was described as an advanced search engine that consolidated information and provided detailed answers. They noted that the tool’s responses were tailored based on previous interactions, enhancing understanding when placed in a relevant context.

*“I also find it time saving/…to translate, look up terms and bounce ideas, and find information on various topics. It’s all gathered in one place, making it more convenient that way.” (2.2).*

GAI was perceived as a study tool, informants noted that computer use was integral to their studies, and GAI complemented other digital services. Its use varies based on task complexity, study context and individual needs. Some also used GAI for leisure activities.

*“…I don’t use my computer that much at home, and when I use GAI, it’s usually on the computer and mostly at the University./…I go to it when I feel I need to.”* (1.2).

#### Consequences from a learning perspective

Informants described that using shortcuts in the learning process to quickly transform facts into knowledge could lead to deficiencies in fundamental and relevant basic knowledge. Increased access to easily accessible information, not critically interpreted, could impair learning ability and result in an inability to understand and apply relevant information. Consequently, tasks could be completed without possessing the necessary knowledge, leading to a lack of competence and an inability to independently achieve educational goals.

*“……now I’ve got an answer and then you probably feel satisfied at the moment./Then some time passes, and you’ve forgotten it. It’s just that you don’t get the long-term understanding. So, you can’t connect it in the future because you’ve only solved the task for the course here and now. But maybe you won’t be able to solve it later on.”* (2.1).

#### Critical approach required

It was noted that GAI could produce incorrect answers despite convincing wording, necessitating verification through other sources. Inadequate prompting instructions and insufficient prior knowledge of the subject area could result in irrelevant answers. Furthermore, GAI sometimes did not disclose the origins of the generated information, making it difficult to determine its accuracy.

*“…you have to double-check everything. It’s often very difficult to use GAI in an area you don’t have any prior understanding of, because then you don’t know if it’s correct or not.”* (2.2).

By evaluating the generated information in relation to their prior knowledge and course literature, informants ensured its accuracy and relevance. It was emphasized that a critical approach was crucial to avoid relying on potentially incorrect or incomplete answers.

*“…you need to find some other source to back it up, or, well, you can’t just rely solely on what GAI says. You need to compare it with something else, like books or other sources.” (3.3).*

#### A virtual friend as a study partner and teacher

Students viewed GAI as an interactive virtual friend always available to provide personalized and supportive responses. It functioned like a competent teacher or study partner, offering advice and acting as a sounding board. GAI was also seen as a virtual group member, supporting discussions and introducing new perspectives. There was gratitude for GAI’s role as a reliable guide, primarily aimed at being helpful and supportive.

*“… I see it as a friend./You can also have a discussion (). Why is it like this or why does it happen, and then you get an answer. So, you can bounce ideas off it.”* (1.4).

#### Guidelines and perceptions of GAI usage in education

Informants described clear guidelines for responsible GAI use and encouraged the integration of GAI into studies as positive support for its use as an educational resource. Organizational integration of GAI, training, and access to models increased confidence, which resulted in GAI being viewed as a valuable learning resource.

“… it is good that there is acceptance to use GAI. Instead of it being seen as cheating, it is seen as a resource…” (2.4)

Informants expressed uncertainty about what was permissible and what constituted cheating when GAI could generate text for assignments. They described a fear of misusing GAI.

*“You’re a bit afraid to use it because you’re afraid of using it incorrectly./So, it feels a bit unclear what you can and can’t do, so I have somewhat mixed feelings about it.”* (2.3).

An ambiguity about acceptable GAI usage emerged.

*“…and that’s where the concern comes in, since some texts I use are written by me but then rephrased by GAI, and then you don’t know where the boundary is.”* (2.4).

Further, the design of most examination components primarily ensured that GAI could not influence the outcomes, leading to the tool being viewed more as enhancing learning rather than as a tool for cheating in studies.

### Future with GAI in education and profession

#### Opportunities and risks associated with GAI development

Informants described the reliability of GAI models, and that the functionality is expected to improve with technological advancements. It was highlighted that GAI is likely to significantly impact various societal sectors due to increased access to information. There were also comments that higher education should adapt and integrate GAI technology rather than impose bans, to align with societal development.

“… GAI will be something that will be used a lot in the same way we’re using it now. But maybe you won’t need to be as critical of it anymore because it becomes more and more reliable.” (2.4).

Concerns were raised that companies developing these technologies could gain excessive power to influence information based on profit interests. Additionally, there was a perceived risk that future generations might lack knowledge equivalent to today in case of over-reliance on future GAI.

*“I think that children and youths today might () not have as much experience with reading or expressing themselves in this way.”* (3.1).

#### Development in medical and healthcare programs

Students believed that there was significant potential for the development of GAI models with subject-specific content, which could improve the quality of the information generated based on evidence-based knowledge. They described that advanced GAI models could lead to more accurate and reliable educational tools, enhancing the overall learning experience. Potential applications include simulating patient cases and providing feedback on practical skills, such as structuring a patient history and clinical examination. An adequate introduction to GAI and a positive attitude from educational institutions are crucial for future implementation, including updating study materials and supplementing regular teaching to promote deep learning.

*“… for example, the combination of GAI and a medical database/you could use it to find () evidence for things or how strong the evidence is, or whether there is any evidence at all…”* (2.3).

#### Potential to contribute to the development of the profession

In the physiotherapy profession, informants perceived GAI could enhance efficiency in record management, freeing up time for patient care. GAI was also seen as a support in decision-making regarding treatment measures and enabling the development of profession-specific models to support evidence-based approaches and adapt to society’s digitalization.

*“…in the future, we will see GAI increasingly integrated into the medical record systems. Partly helping us write records but also providing significant assistance in identifying possible conditions and determining the next treatment measures.”* (1.5).

## Discussion

The main findings were that physiotherapy students perceived GAI as supporting their learning process in various ways, GAI was seen as a virtual friend, as a study partner and teacher and as having the potential to improve education and profession. Another main finding was the students’ experiences and thoughts about disadvantages, such as technological limitations, risks of over-reliance on the tool and uncertainties about what constitutes permitted GAI usage within the academic context. The study findings can be related to the self-regulated learning model, which includes three phases 1) forethought, 2) performance and 3) reflection [12]. The first phase, the forethought phase, involves planning goals and strategies (intentionality) for the learning task and is influenced by the student’s belief in their abilities (self-efficacy). The second phase, performance, focuses on implementing and monitoring these strategies. The third phase, self-reflection, involves reflecting on and adjusting strategy and goals. Therefore, if students believe in their abilities, plan learning goals and strategies, implement them, and reflect on their learning, their student agency will be high.

### Students’ forethoughts on the use of GAI

Students describe using GAI at various stages of the learning process. Initially, GAI was used for an introduction to and inspiration for new subjects. As learning progresses, GAI simplifies complex concepts, by facilitating the understanding of course literature. At more advanced levels, GAI is used to practice clinical reasoning and problem-solving. This is an important finding, as developing clinical reasoning competence is essential for optimizing patient-centered care [34].

Students highlighted GAI’s role as a ‘sounding board’ for clinical reasoning by simulating patient cases and providing feedback. This aligns with research indicating that GAI facilitated learning for medical students by enabling scenario generation and case simulation, providing rapid information, easy to use and explaining complex information [35]. Further, healthcare students emphasized the importance of risk perceptions, usefulness, ease of use, attitudes toward technology, and behavioral factors when adopting ChatGPT in healthcare education [4]. In medical schools the technical aspects of GAI literacy need to be enhanced, there is a positive correlation between AI literacy and positive attitudes toward GAI [36]. The students need to understand the importance of fact-checking GAI information, e.g. when using GAI to assist their clinical reasoning by generating patient cases with typical symptoms and responses to clinical tests. The student’s approach to using GAI is crucial for their success in learning. When students actively question GAI outputs, relate them to their prior knowledge, and consider how these outputs help them achieve an understanding of the subject and their learning goals, they demonstrate high student agency [12, 13].

### Performance control: engagement, attention and willpower

Time-saving and tailored studies through faster information retrieval supporting decision-making and improved wording were highlighted as advantages of GAI usage. This aligns with previous studies emphasizing GAI’s role in facilitating information processing, improved access to information, person-centered information delivery, and academic writing improvements in medical education [5, 9]. The use of GAI may facilitate deeper learning, provided the goal is to facilitate learning and not to take shortcuts [13].

Students described the risk of surface learning, noting that GAI could offer shortcuts, allowing them to complete tasks without fully understanding or applying relevant information. This aligns with previous research showing that easily accessible information, if not critically interpreted, can lead to insufficient understanding [37]. Excessive use of GAI could hinder critical thinking and decision-making, risking dependency on GAI for knowledge acquisition, which is findings in medical students [8] and nurse students [9, 23]. This issue may stem from the knowledge gap between real and AI-generated information [9]. Therefore, developing critical thinking is essential to prevent superficial learning, ensuring students can analyze, evaluate, and apply knowledge to new situations, avoiding deficiencies in competence [37].

It emerged that there was uncertainty about what constitutes permitted use of GAI. This aligns with a survey on attitudes toward AI tools among 5894 Swedish students in their first, second and third year across five different universities, which revealed a lack of clarity in institutional guidelines and the need for clear guidelines for GAI [14]. In this qualitative study, students expressed uncertainty about how using GAI is permitted. While clear guidelines are necessary, they may not fully resolve this uncertainty. The findings suggest a need for supportive learning activities with feedback to enhance students’ self-efficacy and strategies in using GAI. To enhance and optimize students’ GAI usage, it is important to promote high student agency [13].

### Self-reflection in GAI usage

Students described that a critical approach is required due to GAI’s limitations. It is an important finding that students have developed strategies for critical thinking, such as using adequate prompts and verifying GAI results with other sources when responses might be incorrect or misleading. When students set learning goals and strategies while considering GAI’s limitations, they use their metacognitive abilities. This process culminates in the final phase of self-regulated learning ‘reflection’. Self-regulated learning facilitates a deep learning approach. However, if students neglect reflection, they will not achieve this depth. Therefore, fostering a habit of reflection is crucial for maximizing the benefits of self-regulated learning.

Additionally, regarding the approach to GAI, students likened GAI to a friend, study partner, and teacher, always available to help, which provides security in studying. This aligns with Kim et al. [38], showing GAI as an effective learning support, providing continuous feedback and emotional support. It helped students build confidence by breaking down larger tasks into more manageable parts and a gratitude to GAI for acting as a guide and sounding board was expressed [13, 38].

### Supporting responsible GAI use in education

The students emphasized the need for clear guidelines and encouragement from educational organizations to support responsible GAI use today and to keep pace with future technological developments. Previous research shows that GAI can enhance students’ performance, motivation, and learning efficiency. However, it also underscores the need for AI education for teachers and students. Providing technical and pedagogical training can facilitate the ethical and effective integration of GAI into teaching [39].

### Use of GAI in physiotherapy practice

Students anticipated that the development of GAI would have several implications for clinical practice soon. They believed that GAI could support decision-making processes and enhance efficiency in record management, thereby freeing up time for patient care. This expectation aligns with a recent study that found health professions students described GAI as providing quick access to evidence-based guidelines, enhancing knowledge and improving and saving time in clinical decisions [40]. Recent research has shown that GAI holds significant potential for enhancing physiotherapy practice. Tailored GAI applications can analyze patient data, recommend personalized treatment plans, and conduct clinical simulations [17]. Adapting GAI in physiotherapy education involves collaborating with other education settings and incorporating GAI in teaching and clinical experiences, such as using LLM in learning tasks [41]. LLMs could also be used for administrative tasks, simulated patient interactions, clinical decision-making support, guideline recommendations, and tailed treatments [19]. Additionally, computer vision and machine learning can accurately monitor home exercise movements [20]. GAI could be a valuable educational tool for patients, though current models need improvements in accuracy and readability [42]. Despite these advancements, challenges such as ethical considerations and the risk of over-reliance on AI must be addressed to fully leverage the benefits of GAI in physiotherapy practice.

### Strengths and limitations

This study is part of ongoing efforts to conduct educational adaptations in relation to GAI and enhance AI literacy in students and teachers within the Physiotherapy program at Linköping University, Sweden. The students’ experiences and thoughts on GAI in their education provide valuable insights into implementing updated learning activities. Focus groups capture diverse experiences [25, 27], and a series of focus groups increase confidence in the emerging patterns [25]. Group dynamics could influence the conversation, so participants were instructed not to interrupt each other and were asked if they had anything more to add after each question. This approach fostered a good discussion climate and often elicited additional information.

The three focus groups meet the requirement for sufficient analysis [27]. The four to six informants per group are within the ideal size, and dividing informants into homogeneous groups based on term affiliation increases security and fosters open discussions [27]. Further, Patton described that having a small number of participants per group can be advantageous when there are many experiences or strong feelings about the phenomenon studied [25]. However, the low total number of participants may limit the representation of experiences.

We aimed to recruit both GAI users and non-users. However, when interpreting the study findings, it should be noted that students who agreed to participate might be more positive and experienced with GAI than the general student population.

A methodological limitation was that the moderator and assistant were the students’ teachers. This relationship could lead students to avoid discussing sensitive GAI use, affecting truthfulness. To address this, the interview guide was created to encourage honest answers, and thereby enhancing the study’s credibility [25]. Research shows power dynamics can lead participants to modify responses or avoid sensitive topics, especially if they feel subordinate [43].

### Implications of GAI integration in physiotherapy education

The study highlights GAI’s potential to enhance the learning process for physiotherapy students, suggesting that it could improve education and profession in accuracy, and healthcare delivery. However, technological limitations, risk of over-reliance, and uncertainties about proper usage underscore the need for enhanced AI literacy in courses.

These findings emphasize the importance of developing comprehensive curricula that effectively incorporate GAI, ensuring students are well-versed in its advantages and limitations, for their studies and to be prepared for their profession where GAI is integrated. Ensuring access to GAI tools is crucial for fairness and addressing ethical concerns related to academic integrity [23]. To effectively harness the technology, it is essential to develop guidelines that promote innovative teaching strategies and assessments [44].

Learning activities with feedback on GAI usage are essential to support self-efficacy in self-regulated learning, thus maximizing benefits while mitigating risks. Additionally, students need to develop their critical thinking skills and understand the importance of verifying the accuracy of GAI information.

Future research should focus on exploring the long-term impacts of GAI on learning outcomes and professional competencies and investigating strategies to address challenges and ethical concerns associated with its use. Additionally, expanding studies to include diverse student populations and disciplines will provide a more holistic understanding of GAI’s role in education.

## Conclusion

Physiotherapy students found GAI beneficial for learning and clinical reasoning but expressed concerns about its impact on learning quality. They emphasized the importance of a critical approach when using GAI and the need for organizational support, including supporting permitted GAI use. Students believed that future advanced GAI models could provide accurate and reliable educational tools and healthcare tools supporting documentation and evidence-based decision-making. However, potential risks include business profit motives and knowledge gaps. Navigating these challenges is essential to fully leveraging GAI’s benefits in education and physiotherapy practice. Therefore, fostering a critical approach and ensuring robust organizational support is crucial for maximizing the positive impact of GAI in physiotherapy.

## Supplementary Information

Additional file 1.

## Data Availability

All data relevant to the study are included in the manuscript or uploaded as supplemental information. The interview guide is available in Appendix.

## Abbreviations

AI: Artificial Intelligence
ChatGPT: Chat Generative Pre-trained Transformer
COREQ: COnsolidated criteria for REporting Qualitative research
GAI: Generative Artificial Intelligence
IQR: Interquartile range
LLMs: Large Language Models
