# Supplementary material for: Generative artificial intelligence in physiotherapy education: great potential amidst challenges- a qualitative interview study
Source: BMC Med Educ. 2025 Apr 24;25:603. doi: 10.1186/s12909-025-07106-w (PMC12020151; doi:10.1186/s12909-025-07106-w)
Supplement: Supplementary file 1 — Additional file 1. [file 12909_2025_7106_MOESM1_ESM.docx]

GENERATIVE ARTIFICIAL INTELLIGENCE IN PHYSIOTHERAPY EDUCATION: GREAT POTENTIAL AMIDST CHALLENGES- A QUALITATIVE INTERVIEW STUDY

# **Appendix 1**

# **Interview Guide**

1. Would you like to start by sharing what comes to mind when you hear generative AI? The term ChatGPT?
   - In what contexts have you encountered generative AI, such as ChatGPT?
   - What are your general experiences with generative AI, such as ChatGPT?
2. What are your thoughts on using generative AI in your university studies?
3. Would you like to share your experiences of using generative AI, such as ChatGPT, in your university studies?
4. What does generative AI do that makes you choose to use this technology? What purpose does generative AI serve for you?
   - How do you use generative AI? Can you provide examples?
5. How do you perceive the usability of the technology?
6. What potential applications do you see for generative AI, such as ChatGPT, in the physiotherapy program?
   - In what ways do you see generative AI complementing other forms of teaching?
   - In which teaching activities do you think generative AI would be particularly useful?
7. What challenges do you see with using generative AI in your studies?
   - What are your experiences and thoughts on the quality of AI-generated information?
8. What do you think has been the most important topic we discussed today?
9. Take a moment to reflect—is there anything else you would like to share about your experiences and thoughts on the use of generative AI in your studies?
   Are there any additional aspects of our discussion that you feel we may have missed and that you would like to comment on?

**Examples of questions to encourage elaboration and deepen responses in all parts of the interview:**

*Would you like to share more?*

*Would you like to explain that?*

*Could you clarify your point a bit more?*

*I'm not quite sure I understand; could you explain it differently?*

*You mentioned [repeat what they said]; could you expand on that?*
